# Rapeseed Meal as a Sustainable Source of Proteins, Peptides, and Phenolics: Composition, Interactions, and Functional Potential

**DOI:** 10.3390/foods15111930

**Published:** 2026-05-29

**Authors:** Rehman Sarwar, Yixiang Song, Yao Zhang, Xiaoli Tan, Yuanxue Liang

**Affiliations:** 1 School of Life Sciences, Jiangsu University, Zhenjiang 212013, China; rehman_sarwar@outlook.com (R.S.); songyixiang0202@163.com (Y.S.); 18225980845@163.com (Y.Z.); xltan@ujs.edu.cn (X.T.); 2 School of Agricultural Engineering, Jiangsu University, Zhenjiang 212013, China

**Keywords:** *Brassica napus*, plant-based nutrition, bioactive peptides, antioxidant

## Abstract

Rapeseed meal (RSM) represents one of the most abundant yet underutilized alternative plant protein sources, offering a compelling nutritional profile and diverse bioactive compounds with direct relevance to human nutrition and health. Its major storage proteins, including 12S globulin cruciferin and 2S albumin napin, along with phenolic compounds, have been associated with antioxidant, antihypertensive, and potential antimicrobial activities. Beyond these health-promoting properties, RSM-derived storage proteins are emerging building blocks for nanocarriers, with potential applications in drug delivery systems. Recent studies have advanced extraction, purification, and modification strategies for RSM bioactive compounds. However, relatively little attention has been given to how specific molecular interactions between phenolics and proteins modulate functional properties and bioactivity. This review, therefore, provides an updated analysis of the bioactive profile of RSM bioactive compounds, the associated phenolic–protein interactions, and their functional potential. Additionally, we also discuss the current challenges and future opportunities for integrating RSM bioactive compounds into biotechnology and multi-product biorefinery schemes.

## 1. Introduction

Proteins are fundamental biological macromolecules required to perform essential functions in living organisms, including catalyzing biochemical reactions, transporting and storing molecules, mediating cell signaling, and regulating growth, differentiation, and immune responses. Driven by global economic development and increasing populations, the intake of meat and other animal-source foods has increased, which has resulted in the excessive usage of animal-based protein resources, placing pressure on the environment [1]. In comparison, plant-based protein sources offer important advantages in environmental impact and certain health outcomes, including lower greenhouse gas emissions and saturated fat intake and the absence of dietary cholesterol [2]. When appropriately formulated, plant proteins can provide adequate nutritional quality while also serving as carriers of diverse bioactive compounds that contribute additional antioxidant, anti-inflammatory, and cardiometabolic benefits. Combined with their lower environmental impact, potential health advantages, and broad functional and industrial applications, these features have made plant proteins and their associated bioactivity a major focus of contemporary food science and nutrition research. At the same time, many plant-based proteins face important nutritional challenges, including generally lower digestibility in their native forms; the presence of antinutritional factors; and imbalances in indispensable amino acids, particularly lysine and sulfur-containing amino acids and, in some cases, threonine, which can limit their standalone protein quality relative to animal proteins [3].

Rapeseed (*Brassica napus*) is a cool-season oilseed crop widely cultivated in temperate regions across Europe, Canada, China, and India. More recently, global rapeseed production has reached approximately 88 million metric tons, making it the second-largest oilseed crop worldwide [4,5]. Rapeseed yields both high-quality edible and industrial oil and a protein-rich meal, which makes it a major global source of vegetable oil and plant protein [6]. Rapeseed meal (RSM), the principal by-product of rapeseed oil extraction, is mainly used as a protein source in animal feeds and is the second most important protein meal after soybean meal [7]. RSM provides a high crude protein content around 35–45% (*w*/*w*) depending on cultivar and processing, with a well-balanced amino acid composition comparable to soybean meal. In particular, RSM is characterized by relatively elevated levels of sulfur-containing amino acids, especially methionine and cysteine, and lower lysine content, which allows it to complement the amino acid deficiencies of cereal-based feeds and foods [8,9,10]. Compared with other plant-based protein meals, RSM is generally cheaper than soybean meal and is further characterized by high-levels of choline, niacin, and several B vitamins, as well as elevated amounts of minerals including calcium, phosphorus, selenium, iron, and manganese, which enhances its value as a nutrient-dense ingredient in compound feeds [8,11,12]. Additionally, RSM contains substantial quantities of phenolic compounds, which exhibit pronounced antioxidant activity and can be recovered as natural antioxidant ingredients for food, cosmetic, and pharmaceutical applications [13,14,15]. In practice, rapeseed proteins and rapeseed-derived isolate are best positioned as components of complementary plant protein blends, where their strong sulfur amino acid content, mineral and vitamin profile, and functional properties are combined with lysine-rich sources such as pulses and cereals to optimize overall protein quality for both animal feeds and human food.

Moreover, following protein extraction, the residual RSM becomes relatively enriched in lignocellulosic polysaccharides, which can serve as a fermentable carbon source for microbial production of biopolymers and other bio-based chemicals [16,17]. However, the use of RSM in livestock and aquafeeds is constrained by antinutritional factors such as glucosinolates, phytic acid, and high fiber, which reduce energy availability and amino acid digestibility [18]. Recent advances in plant breeding, processing, as well as biological treatments such as microbial fermentation and phytase supplementation, have enabled the use of RSM as a partial replacement for conventional protein sources in animal feeds [8].

In addition, RSM protein isolates exhibit favorable emulsifying and gel-forming properties, along with processing-dependent antioxidant activity, which support their use as multifunctional ingredients in foods [12]. They have been incorporated into products such as gluten-free breads, plant-based beverages, and texture-modified foods, where they can enhance protein content, modulate texture, and contribute to improved oxidative stability of lipid-containing systems [11,19]. Beyond food applications, rapeseed proteins and their derivatives have recently emerged as versatile building blocks for advanced biomaterials [12,20].

Despite a historical tradition of utilization as a protein ingredient and promising nutritional profile, the behavior of its phenolic-rich and protein-rich fractions, and the molecular interactions between endogenous phenolics and storage proteins, remains poorly characterized. The mechanisms by which these interactions shape techno-functional properties and biological activities are still unclear, limiting detailed characterization of RSM bioactive and the development of high-value applications beyond conventional feed uses. This review provides an up-to-date overview of the composition of RSM bioactive compounds and synthesizes current knowledge on their distinctive properties, molecular interactions, and resulting functional and biological activities. In contrast to earlier reviews, which mainly address nutritional quality, bulk functional properties, and general applications of canola/rapeseed proteins and meal [21,22,23], this work offers a systematic, interaction-focused synthesis linking the detailed molecular composition of RSM proteins, peptides, and phenolics with protein-phenolic interaction mechanisms across processing operations, as well as the resulting techno-functional properties and bioactivities in food and biorefinery contexts. Furthermore, it introduces a cross-cutting perspective on the biological effects of RSM-derived compounds by distinguishing key bioactivities, including antioxidant, antihypertensive, antimicrobial, and anti-inflammatory, according to their predominant evidence levels (in silico, in vitro, animal, and human studies). Thereby clarifying where evidence remains preclinical and where translational opportunities and research gaps are most pronounced.

## 2. Methods

**Figure 1:**
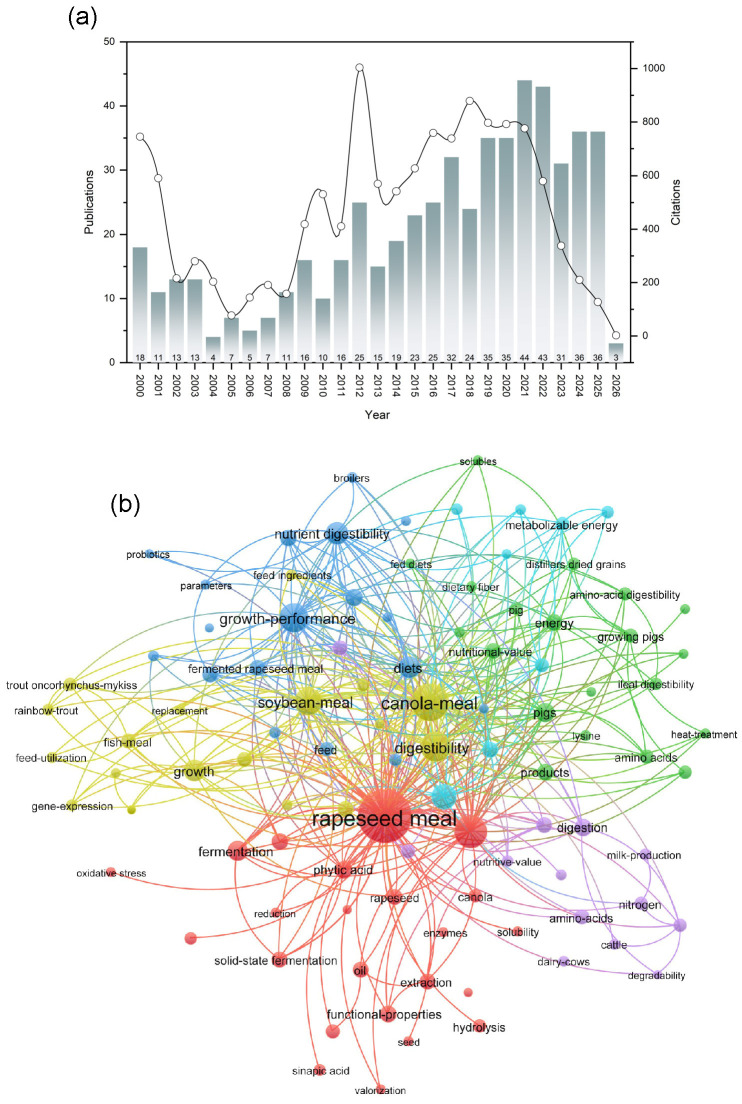
General overview of the publication related to the topic. (**a**) The number of publications and citation frequency on RSM from 2000 to 2026. (**b**) A keyword matching network analysis of publications on RSM.

The literature search was performed electronically in the Web of Science database and yielded 596 documents published between 2000 and 2026. The extracted data were synthesized and critically analyzed to summarize key findings and to highlight remaining research gaps. Figure 1 displays the progress in research on RSM. Overall, the number of publications on RSM has increased markedly since 2000, with particularly strong growth after 2015, reflecting rising interest in alternative proteins, side-stream valorization and biorefinery concepts. Keyword network analysis (Figure 1b) highlights clusters around ‘rapeseed meal’, ‘protein’, ‘phenolics’, ‘antinutritional factors’, ‘extrusion’, and ‘fermentation’, indicating parallel developments in compositional, techno-functional, and processing research, which are discussed in the following sections.

## 3. Bioactive Compounds of RSM

### 3.1. Phenolic Compounds

#### 3.1.1. Sinapine

Sinapine, a quaternary ammonium ester of sinapic acid (sinapoylcholine), is the predominant polar phenolic compound in RSM. Collectively, sinapate esters account for up to 2% of seed dry matter, with sinapine typically representing 70 to 90% of extractable phenolic fractions [13,24]. It is largely characteristic of plants in the *Brassicaceae* family, in which sinapate esters represent the major phenylpropanoid reservoir [25,26]. The chemical diversity of sinapic acid derivatives in rapeseed is substantial, with more than a dozen distinct esters and conjugates identified to date [15,27], including sinapoylglucose and 1,2-disinapoylgentiobioside as prominent esters, as well as minor components such as sinapoylmalate, feruloylcholine, and various kaempferol-sinapate glycosides [13,25,28]. However, the distribution of these derivatives within rapeseed is not uniform. The cotyledons and dehulled meals are particularly enriched, with sinapine in processed meals typically ranging from about 5 to 35 mg per gram dry matter, whereas free sinapic acid is generally present at 0.1 to 5 mg per gram, depending on cultivar and processing conditions [27].

Functionally, sinapine and other sinapate esters display antioxidant potential; however, their activity generally appears lower than that of free sinapic acid, likely because of esterification-induced changes in the ionization state, which restrict the availability of phenolic hydroxyl groups for hydrogen atom transfer and single-electron transfer reactions [13,15,29,30]. Despite these promising bioactivities, practical exploitation of sinapine is limited by moderate stability and uncertain oral bioavailability. High-temperature processing, pressurized extraction, and roasting can promote hydrolysis of sinapate to sinapic acid and canolol, causing substantial compositional shifts if processing is not carefully controlled [31,32]. In vitro digestion models, cellular transport, and microbiota-focused studies indicate extensive transformation of sinapate esters during gastrointestinal transit and suggest that gut fermentation may modulate their antioxidant and anti-inflammatory activities [13,24,27,29,31], although sinapine-specific in vivo pharmacokinetic data remain lacking.

#### 3.1.2. Flavonoids

RSM has also emerged as a promising reservoir of flavonoids, which underpin diverse pharmacological properties. Previously, a study by Oskoueian, et al. [33] reported that methanolic extracts of RSM contain higher total flavonoids and show stronger inhibition of lipid oxidation than cottonseed or soybean meals. Recently, targeted and untargeted LC-MS-based metabolomics have greatly expanded the known flavonoid spectrum in RSM, including kaempferol, quercetin, isorhamnetin, astragalin, and gossypin [34,35]. In addition to these conjugated flavonoids, RSM also contains minor amounts of aglycones, whose profiles vary between yellow and black seeded genotypes and across environments [36,37]. These compositional data support the view of RSM as a structurally diverse flavonoid source.

This diversity translates into pronounced antioxidant, anti-inflammatory, and enzyme-inhibitory properties. For instance, recent studies have revealed that methanolic extracts rich in flavonoids from RSM display strong DPPH and nitric-oxide radical scavenging activities, xanthine oxidase and tyrosinase inhibition, indicating potential application as natural antioxidant agents [35,36,37]. In addition, flavonoids, such as quercetin and kaempferol from rapeseed, have also been investigated for their capacity to modulate inflammatory signaling, lipid peroxidation, and mitochondrial pathways, broadening functional mechanisms for the cardiometabolic relevance of rapeseed-derived flavonoids [34]. Despite these advances, many flavonoids in RSM remain only tentatively annotated. Thereby, combining high-resolution LC-MS with molecular networking is essential to quantify key flavanol, flavanone, and flavanol glycosides in RSM. Taken together, these findings indicate that RSM flavonoids, encompassing both simple and highly glycosylated structures, represent a valuable yet underexploited resource with promising applications in functional foods and high-value biorefinery applications.

In addition to these compounds, chlorogenic acid and other phenolic acids are also present in RSM, mainly in esterified and insoluble-bound forms [38]. Phenolic acids at high concentrations have been associated with reduced protein digestibility and bioavailability through complex formation with proteins and digestive enzymes. However, current data suggest that the overall limitation of protein digestibility in RSM is driven by the combined effects of total phenolics, fiber, phytate, and other antinutritional factors, rather than chlorogenic acid alone [39,40], and the specific contribution of chlorogenic acid at its naturally occurring levels in RSM remains to be quantified.

### 3.2. Storage Proteins

**Figure 2:**
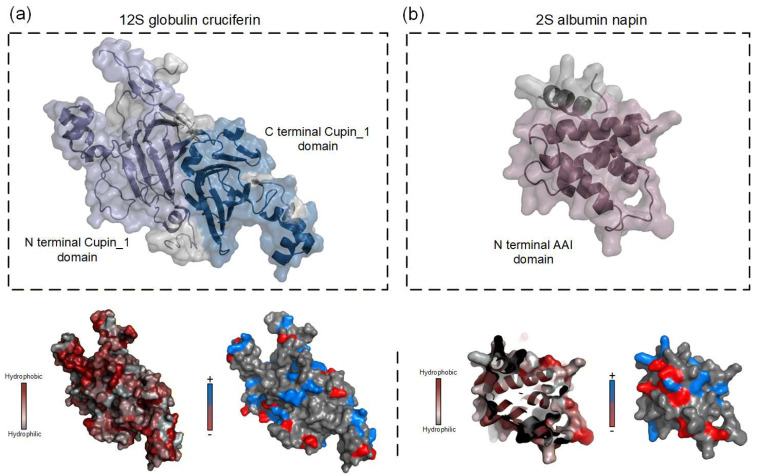
Storage protein structure of rapeseed protein. (**a**) Crystal structure of 12S globulin cruciferin (PDB:3KGL). Each cruciferin monomer contains two paralogous cupin domains, an N-terminal and a C-terminal cupin domain, each adopting the conserved β-barrel fold characteristic of the cupin superfamily. The inter-domain regions between cupin domains serve as primary cargo encapsulation sites, and the large surface area provides multiple weak-affinity binding sites for hydrophobic compounds. The lower panel displays pronounced hydrophobic characteristics, with large continuous patches of nonpolar residues exposed on the protein surface that facilitate protein aggregation and molecular packing. (**b**) Crystal structure of 2S albumin napin (PDB:1PNB), displaying the N terminal albumin allergen inhibitor (AAI) domain. The right lower panel demonstrates a more balanced electrostatic profile with interspersed hydrophobic and hydrophilic patches, consistent with its compact structure and high stability.

RSM is characterized by a higher quantity of storage proteins which, in combination with phenolic compounds, facilitate antioxidant and anti-inflammatory properties. Proteomic analysis has identified more than 200 different proteins in RSM, with the two major storage proteins being the 12S globulin cruciferin, which accounts for 60%, and the 2S albumin-type napin, contributing about 20 to 40% [41,42,43]. Cruciferin is the main globulin in RSM, with a molecular weight of 300 to 350 kDa and a hexametric structure of six subunits arranged as two trimers [44,45,46]. Crystallographic and biophysical characterization revealed that each cruciferin subunit is derived from a single procruciferin precursor that is post-translationally cleaved into an α-polypeptide of 30 kDa and a basic β-polypeptide of 20 kDa, which remain covalently associated via a single interchain disulfide bond, yielding a mature subunit of approximately 50 kDa. Recently, recombinant studies on pro-cruciferin isoforms confirm trimer formation and demonstrate that proteolytic processing induces hexamer stabilization [45,47]. Cruciferin belongs to the cupin superfamily, whose members share a conserved β-barrel cupin domain (Figure 2), involved in stable packing and storage of amino acids [41]. Each monomer contains two cupin domains that form a compact β-barrel core (Figure 2). In contrast, napin is a basic 2S albumin with a molecular weight of approximately 12 to 16 kDa. In RSM, napin is typically present as several isoforms that share a high degree of amidation and a conserved disulfide-bonding pattern [44,48,49,50]. Each napin isoform contains four conserved disulfide bonds, two interaction bonds linking the short and long polypeptides, and two intrachain bonds within the long chain, which collectively stabilize the compact helical fold and create defined surface-exposed loop regions with conformational flexibility (Figure 2).

These two major storage proteins have markedly different amino acid compositions and structural functional characteristics, with napin being richer in basic residues (arginine, lysine, histidine) and cruciferin exhibiting a more β-sheet-dominated globular architecture (Figure 2) [41]. Napin is generally considered more soluble across a wide pH range, whereas cruciferin shows pronounced pH-dependent solubility and structural unfolding, particularly at low pH. The distinct amino acid composition, net charge, and quaternary structure of cruciferin and napin likely underpin their contrasting interfacial and bulk functional properties in RSM proteins [19,51,52]. At most pH values, napin, owing to its higher solubility and smaller molecular size, contributes strongly to emulsifying capacity, whereas cruciferin forms stiffer, more elastic interfacial layers that enhance foam and emulsion stability against coalescence, particularly at acidic to neutral pH, rather than markedly increasing the maximum oil fraction that can be incorporated [51,52]. Comparable complementary behavior between globulin-type and albumin-type storage proteins has been observed in rapeseed protein systems where varying cruciferin-to-napin ratios allow tailoring of foam half-life, emulsion stability, and emulsion-filled gel firmness by balancing napin-driven interfacial adsorption with cruciferin-driven interfacial rigidity and bulk gelation [19,51,52]. Current studies on rapeseed protein systems with varying cruciferin and napin ratios show such complementary behavior but do not yet clearly establish whether cruciferin can stabilize emulsions at substantially higher oil contents than napin [51,53]. RSM also comprises a minor but functionally significant fraction of oil body proteins, including oleosins, caleosins, and steroleosins, whose amphiphilic domains and integration with native oleosomes contribute to stable oil droplets and enhance the interfacial activity and storage stability of oleosome protein complexes [44,45].

Beyond their technological and bioactive functions, rapeseed storage proteins also raise allergenicity concerns. Napin has been identified as a major seed allergen in children sensitized to oilseed and turnip rape, and napin genes in *Brassica rapa* encode proteins with conserved IgE-binding epitopes typical of 2S albumin allergens [54,55], although clinical reactions to rapeseed in mustard-allergic patients have not yet been clearly documented [56,57]. In addition, RSM comprises a minor but functionally significant fraction of defense-related proteins, including thionins, trypsin inhibitors, and lipid transfer proteins, which can transiently reduce protein digestibility by inhibiting digestive proteases [42]. However, in conventional RSM, these inhibitors are present at comparatively low levels, and their activity is further reduced by the heat and processing conditions typically applied during oil extraction and subsequent detoxification.

### 3.3. Amino Acid Composition

Compared with other oilseed meals such as sunflower, RSM provides a well-balanced essential amino acid profile, characterized by relatively high levels of sulfur-containing amino acids and adequate branched-chain amino acids (BCAAs) (Table 1) [58], while some non-essential amino acids, such as glycine and proline, occur at moderate levels [58]. Overall, RSM proteins provide a favorable amino acid profile, with high levels of sulfur-containing amino acids and a slightly lower lysine concentration, but similar or higher levels of methionine, cysteine, and certain aromatic amino acids, enabling their complementary use in mixed feeds and formulated foods to improve amino acid balance [42,58,59]. In practical formulations, lysine (and in some cases threonine) can therefore act as a potentially limiting amino acid when RSM is used as the sole protein source, which underlines its optimal use in combination with lysine-rich ingredients such as pulses and cereals. Recent human and animal studies indicate that rapeseed proteins and cruciferin-rich isolates can achieve high digestible indispensable amino acid scores (DIAAS) with no limiting indispensable amino acids when properly processed [60,61,62]. These findings underscore that, although lysine can be limiting in single-source or poorly balanced rapeseed formulations, RSM-derived proteins can meet FAO/WHO amino acid recommendations and requirement patterns when used in well-designed, mixed diets and when processing effects on lysine availability are carefully managed.

The high proportion of umami amino acids, such as glutamic acid and aspartic acid, may support overall flavor intensity, although this effect is often masked by the bitterness associated with phenolic compounds and hydrophobic peptides [21]. RSM is enriched in glutamic acid, aspartic acid, arginine, and hydrophobic amino acids, while albumin-napin-derived peptides contribute comparatively high levels of sulfur-containing residues and lower proportions of aromatic residues, reflecting the characteristic amino acid profile that underpins both the nutritional quality and sensory behavior of rapeseed hydrolysates [42,63]. Recently, it has been reported that peptide-rich rapeseed protein concentrates and hydrolysates exhibit bitter and astringent tastes, especially at medium to high degrees of hydrolysis. This effect is especially pronounced in untreated or fermentative systems, where the formation of abundant short hydrophobic peptides coincides with increased bitterness [64,65]. However, relatively few rapeseed-derived peptides have been structurally resolved and directly linked to bitterness [64]. For instance, Spaccasassi et al. [64] indicate that short hydrophobic peptides act with polyphenolic kaempferol glycosides, such as kaempferol 3-O-(2′-O-sinapoyl-β-D-sophoroside) (K3OSS), act as dominant bitter and astringent drivers in many rapeseed isolates (Table 1).

**Table 1:** Proximate macronutrient composition and amino acid profile of RSM.

Constitute	Compounds	Quantity	References
Proteins, fats and carbohydrates (%)	Total Protein	35–45	[8,9]
	Carbohydrates	29–54.7	
	Fat	2.8	
Essential amino acids (g/kg)	Isoleucine	15.3–38	[8,9,66,67]
	Valine	19.7–52	
	Threonine	17.6–45	
	Lysine	19.5–58	
	Leucine	27–66	
	Methionine	7.6–19	
	Histidine	10.1–27	
	Phenylalanine	15.3–37	
	Tryptophan	5.1–13	
Non-essential amino acids (g/kg)	Serine	41	[8,66,67]
	Aspartic acid	71	
	Proline	60	
	Glycine	48	
	Glutamic acid	173	
	Tyrosine	25	
	Arginine	58	
	Alanine	43	
	Cysteine	24	

Moreover, recent work on rapeseed fermentations for high-moisture meat substitutes, including burger and fillet-type analogues, shows that uncontrolled proteolysis by endogenous or microbial proteases generates abundant short peptides that increase bitterness and markedly impair the formation of fibrous structure required for realistic bite and juiciness, underscoring the need to fine-tune fermentation conditions and peptidase specificity for such products [65,68]. Conversely, process optimization studies on rapeseed protein hydrolysates indicate that combined enzymatic strategies with balanced endopeptidase and exopeptidase activities, together with prior ethanol and salt based dephenolization, favor the generation of low-bitter, umami-forward plant protein hydrolysates [69]. Furthermore, the studies on pea proteins suggest that the presence of Glu and Asp-rich anionic oligopeptides, together with Arg- and Lys-containing sequences, can shift the taste profile toward greater umami and saltiness while diminishing bitterness. However, the specific RSM amino acid profile responsible for umami and salt enhancement remains largely uncharacterized, and current efforts focus mainly on phenolic and K3OSS removal [64,70]. Enzymatic hydrolysis may also influence the functional properties of RSM proteins, including solubility, emulsification, and protein interactions important for fibrous structure formation in meat analogs. However, the relationship between hydrolysis and fibrous texture development in RSM-based products remains insufficiently understood and requires further investigation.

Overall, when combined with other lysine-rich plant proteins, RSM protein and its derived peptides provide a relatively well balanced indispensable amino acid profile, making RSM a promising plant protein source for food applications. After ethanol-based dephenolization and optimized extraction or hydrolysis, the peptide profile, digestibility, and antinutrient load can be further tailored, primarily via removal and transformation of phenolic compounds and modulation of peptide size distribution, to yield rapeseed-derived peptide preparations better suited for food processing and other value-added applications. However, substantial challenges remain due to the presence of antinutritional factors, along with fiber and phenolic compounds, which greatly reduce the nutrient bioavailability and functional properties of RSM proteins. Consequently, pre-treatments to remove these antinutritional factors are essential, despite altering the amino acid profile. In addition, genetic factors and agronomic conditions, including variety and nitrogen fertilization, further impact the amino acid composition. Therefore, alternative processing conditions are needed to balance antinutrient removal, protein quality, and functional performance for utilization of RSM proteins.

### 3.4. Other Bioactive Compounds

RSM also contains various other bioactive compounds that enhance its functional and nutritional properties. This includes glucosinolates, specifically 3-butenyl and 2-hydroxy-3 butenyl glucosinolates, which are concentrated in the dry seed coat and residual tissues. They provide a unique pungent bitter smell while exhibiting antimicrobial and potential anticancer properties; however, higher levels possess antinutritional effects [8,71,72,73]. These compounds are typically targeted through pretreatments such as thermal treatment, solvent washing, and enzymatic degradation steps within the detoxification and processing strategies described in Section 7. Additionally, RSM also contains residual lipid components enriched in polyunsaturated fatty acids and minor carotenoids, with linoleic and α-linolenic acids as predominant fatty acids and lutein as the major carotenoid [38,71,74]. These constituents contribute to oxidative stability and may support cardiometabolic health, but residual lipids are also partially removed or re-distributed during the antinutritional-factor mitigation and protein-concentration processes outlined in Section 7, with consequences for both functionality and energy density.

Furthermore, RSM is a valuable source of essential minerals and trace elements, including potassium, phosphorus, calcium, magnesium, iron, zinc, and selenium, alongside B-complex vitamins and choline, which are important for enzymatic function, redox balance, and immune health, although phytic acid can partially limit mineral bioavailability [38,75,76,77]. Finally, RSM with a dietary fiber content typically between 33 and 40% in cold-pressed meals serves as an excellent source of insoluble and soluble fiber [37] that can be utilized as a functional ingredient to promote beneficial microbiota and contribute to the production of short-chain fatty acids and other health-promoting fermentation compounds in the gastrointestinal tract.

## 4. Techno Functional Properties of RSM Bioactive Compounds

### 4.1. Radical-Scavenging Capacity

The presence of phenolic compounds, especially sinapic acid and its derivatives, is the primary source of antioxidant activity in RSM, with other phenolic acids, tannins, and flavonoids providing secondary contributions. These phenolic compounds, which are more abundant in rapeseed than in other oilseed plants, are largely retained within the meal following oil extraction, thus preserving their antioxidant efficacy.

The free radical capacity of the RSM is attributed to its rich profile of bioactive compounds and antioxidant peptides [14,78]. A study by Oskoueian, Abdullah, Hendra and Karimi [33] found that the RSM exhibits a higher antioxidant capacity compared to cottonseed and soybean meals. The measurement of radical-scavenging activity of rapeseed antioxidants involves various methods, each contributing to the understanding of their antioxidant potential. These methods primarily focus on evaluating the ability of rapeseed-derived compounds to neutralize free radicals. Platzer, et al. [79] reported the radical-scavenging activity of RSM, in which the 1,1-diphenylpicrylhydrazyl (DPPH) assay showed the highest correlation with the antioxidant behavior of phenolic compounds in rapeseed. Furthermore, a study by Pan, et al. [80] demonstrated that the rapeseed protein hydrolysates exhibit significant antioxidant activities through various in vitro assays. The rapeseed protein hydrolysates produced via enzymatic hydrolysis with Alcalase show a dose-dependent scavenging effect against free radicals, with notable values for DPPH, superoxide (O2) and hydroxyl radicals (OH). Additionally, rapeseed protein hydrolysates possess a high nutritive value, characterized by a rich amino acid profile, suggesting their potential as a functional food ingredient with health benefits. Furthermore, extraction using supercritical CO_2_ or ethanol further confirms that RSM displays strong DPPH anti-radical activity suitable for food, cosmetic, and pharmaceutical applications [14]. Similarly, further processing via enzymatic and pH treatments yielded RSM protein hydrolysates that effectively neutralize free radicals and inhibit lipid peroxidation [12].

**Figure 3:**
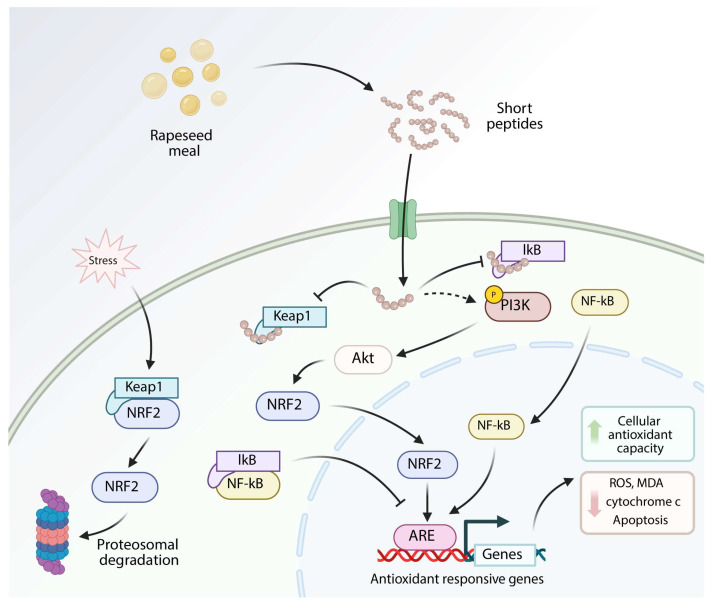
A schematic illustration depicting the roles of RSM-derived bioactive compounds in regulating antioxidant defense mechanisms and associated redox-sensitive signaling pathways. Dashed arrows indicate putative but experimentally unconfirmed interactions for RSM. In particular, the activation of the KEAP1-NRF2-ARE axis and several upstream redox-sensitive kinases are inferred from general polyphenol literature and have not yet been directly demonstrated for RSM proteins and phenolic extracts. This figure was created with BioRender.com (https://www.biorender.com/).

Different peptides have also been reported to show antioxidant properties at the molecular level in scavenging and quenching free radicals. For instance a study by Liu, et al. [81] discovered that the radical scavenging peptide (Pro-Ala-Gly-Pro-Phe) derived from rapeseed protein hydrolysates exhibited significant antioxidant activity for DPPH. It has been shown that the presence of aromatic residues, including phenylalanine (Phe) and cyclic residues such as proline (Pro) in peptides correlates with higher radical scavenging activity, as they facilitate electron or hydrogen donation and stabilization of the resulting radicals through resonance and related delocalization effects [82,83]. Antioxidant peptides from rapeseed protein hydrolysates are often low-molecular weight (<3 kDa) and are enriched in hydrophobic and aromatic residues, thereby contributing in ACE inhibition, anti-inflammatory, and hypolipidemic effects [84,85] (Figure 3). Additionally, peptide fractions isolated from RSM have also shown chelation capacity comparable to ethylenediaminetetraacetic acid (EDTA) and sodium citrate (Na_3_C_6_H_5_O_7_) [42].

Based on the studies of RSM, animal diets can significantly improve the antioxidant status. Hu, et al. [86] described that the feeding of fermented RSM to broilers led to higher levels of serum total antioxidant capacity. Similarly, studies on fish have also indicated that dietary inclusion of RSM, at appropriate levels, did not increase plasma malondialdehyde content and helped maintain the activities of hepatic antioxidant enzymes, including catalase, peroxidase, and glutathione peroxidase. These findings suggest that RSM can help preserve antioxidant defenses in animals [87,88]. Taken together, the high levels of sinapic-acid-based phenolics and bioactive peptides indicate that the radical-scavenging function of RSM is largely driven by its phenolic density and structure, rather than classic vitamins. Despite this potential, the presence of antinutritional factors such as glucosinolates and erucic acid limits its direct application, necessitating further processing to enhance its nutritional value and broaden its utility. However, these antioxidant properties are currently supported largely by in vitro assays and a limited number of animal feeding studies, and controlled human trials with defined RSM ingredients are still missing (Table 2).

### 4.2. Anti-Inflammatory Activity

The anti-inflammatory properties of RSM are primarily associated with its ability to inhibit inflammatory pathways. JNK is a stress-activated mitogen-activated protein kinase that responds to cytokines, pathogens, and oxidative stress to regulate inflammation, apoptosis, and cell survival [89]. During stress conditions, the upregulated expression of *MAP3Ks* and *MAP2Ks* activates JNK, which then phosphorylates AP-1 transcription factors c-Jun, triggering the inflammatory and death response [89]. In parallel, stress signals activate NF-kB and p38-MAPK pathways, which operate independently or concurrently with JNK signaling, depending on cell type, stimulus intensity, and duration of stress exposure. Through this context-dependent crosstalk, NF-kB and p38-MAPK responses limit JNK-mediated ROS amplification and mitochondrial damage (Figure 3). A study by Zhang, et al. [90] demonstrated that RSM extracts and derived peptides can significantly reduce the expression of pro-inflammatory cytokines such as IL-1β, IL-6, and TNF-α by modulating the JNK-independent pathway (Figure 3). Additionally, Han, et al. [91] discovered similar findings, where RSM protein hydrolysates reduced inflammation by decreasing IL-6, IL-1β, iNOS, and COX-2 expression and activating the NF-kB pathway (Figure 3). Moreover, an animal study by He, et al. [92] observed that the rapeseed peptides LY, RALP, and GHS reduced NO, IL-6, and TNF-α, thereby decreasing oxidative stress and inflammatory cytokines in hypertensive rats [90,92,93]. Yeast-fermented RSM extract reduced LPS-induced cytokines and chemokines and oxidative stress markers in Caco-2 and HT29 cocultures, performing comparably or better than ZnO [92,94]. In weaned piglets, dietary fermented RSM decreased intestinal TNF-α, IL-1β, IL-6, and IL-8 and improved oxidative status without impairing growth. Oskoueian, Abdullah, Hendra and Karimi [33] compared rapeseed, cottonseed, and soybean meal biological activities and found that the RSM displayed a superior anti-inflammatory activity [94,95] (Table 2).

Additionally, the antioxidant functionality of RSM may contribute to activation of the KEAP1-NRF2-ARE signaling pathway (Figure 3), although this remains to be demonstrated experimentally. As this supported by extensive evidence that antioxidant-rich functional foods and polysaccharides can promote *NRF2* nuclear translocation and induce antioxidant activity in animal models [96]. Nuclear factor erythroid 2-related factor 2 (NRF2) is a master transcription factor that orchestrates cellular antioxidant and anti-inflammatory responses [24,92]. Under normal conditions, NRF2 is mainly retained in the cytoplasm by Kelch-like ECH-associated protein 1 (KEAP1), which serves as a substrate adaptor for a Cul3-based E3 ubiquitin ligase complex and targets NRF2 for proteasomal degradation, thereby maintaining lower NRF2 activity [97]. However, during oxidative stress, Keap1-dependent ubiquitination of NRF2 is impaired, which allows newly synthesized NRF2 to accumulate and escape Keap1-dependent degradation. NRF2 is then translocated to the nucleus, where it binds to antioxidant response elements (AREs) in the promoter regions of antioxidant responsive genes (Figure 3), thereby enhancing tolerance against oxidative stress. Overall, the anti-inflammatory potential of RSM peptides and extracts is supported predominantly by cell-culture and animal data, and should therefore be regarded as preclinical rather than clinically validated (Table 2).

### 4.3. Antimicrobial Agents

The RSM protein and its hydrolysates are emerging as potential sources of antimicrobial peptides (AMPs). Enzymatic and in silico hydrolysis of the major seed storage proteins napin, cruciferin, and oleosin has generated multiple peptide sequences predicted to possess antimicrobial properties, most of which are cationic, structurally compatible with known AMPs [98]. In particular, in silico digestion of these proteins with food-grade proteases produced 26 novel AMP candidates, with cruciferin yielding more predicted AMPs than napin or oleosin [98]. Complementary in silico proteomic profiling of cold-pressed RSM indicates that thousands of peptides with diverse bioactivities can be generated, although antibacterial activity appears relatively rare and remains largely unconfirmed experimentally [42].

**Figure 4:**
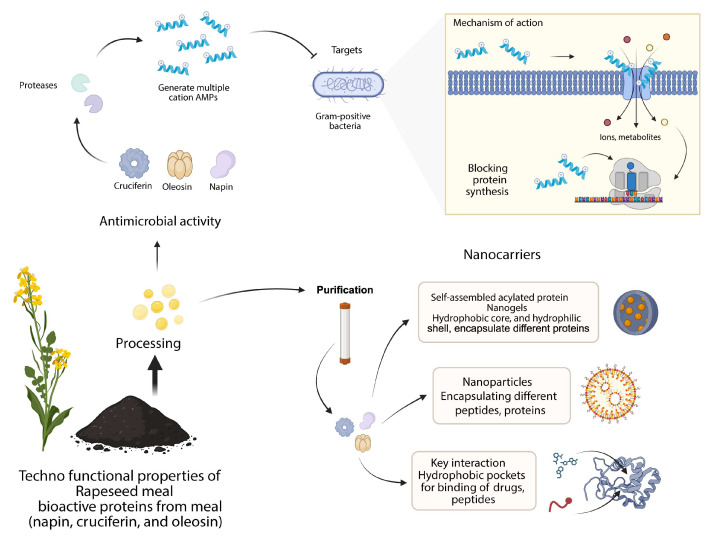
Schematic illustration of the action of RSM bioactive proteins and their nanocarrier formulations. This figure was created with BioRender.com (https://www.biorender.com/).

Experimental evidence for antimicrobial activity of rapeseed proteins is currently restricted mainly to purified napin [99,100]. As previously demonstrated by Rahman, Browne, Van Crugten, Hasan, Liu, and Barkla [48], rapeseed seed storage proteins, particularly the 2S albumin napin, exhibit measurable inhibitory effects against Gram-positive bacteria such as *Staphylococcus saprophyticus* in vitro, whereas cruciferin did not display detectable antimicrobial activity under the same assay conditions. Process engineering studies have shown that controlled proteolysis of total RSM proteins can selectively hydrolyze cruciferin to generate mineral-chelating and antioxidant peptides while keeping napin intact and enriched, creating a protein system that could theoretically provide both oxidative stability and antimicrobial protection in foods [98,100,101]. However, direct demonstration of antimicrobial effects for RSM hydrolysates or napin-enriched fractions in real food matrices remains absent [98,100] (Figure 4).

Based on in silico predictions and limited experimental evidence, the antibacterial activity of rapeseed protein-derived antimicrobial peptides is hypothesized to involve binding to bacterial membranes through electrostatic interactions, leading to membrane permeabilization and intracellular leakage, while some peptides may additionally target nucleic acid and protein synthesis [48,98,99,102]. These proposed mechanisms mirror those established for other food-derived and designed AMPs, where amphipathic, positively charged peptides destabilize membranes and, in some cases, target enzymes or nucleic acid processing machinery. However, direct experimental validation of these mechanisms for most rapeseed-derived sequences remains to be performed.

From an application perspective, rapeseed seed storage proteins and their enzymatic hydrolysates can be considered promising but still largely theoretical sources of plant-derived AMPs for natural food preservatives and antimicrobial agents. Napin-rich fractions, maintained during selective hydrolysis of cruciferin, have been proposed as natural agents to enhance food safety against microbial contamination [101,103]. Additionally, rapeseed protein hydrolysate incorporated into chitosan-based films has been reported to improve film density and antimicrobial performance compared with chitosan alone, suggesting a possible role for RSM peptides in active packaging systems [48,102]. However, most antimicrobial claims for RSM-derived peptides are currently based on in silico predictions and in vitro tests against a limited number of strains, and no published studies have yet demonstrated robust antimicrobial efficacy in complex food matrices or in vivo (Table 2).

**Table 2:** Bioactive properties of RSM-derived compounds, experimental evidence, and reported functional outcomes.

Biological Activity	RSM Bioactive Components	Study Model	Key Functional Outcomes	References
Antioxidant	Phenolic extracts, sinapine, sinapic acid, and protein hydrolysates	In vitro and animal model	Strong in vitro radical scavenging and ACE/renin inhibition; oral intake lowers blood pressure in SHR, suggesting in vivo antioxidant contribution alongside antihypertensive effects.	[88]
Anti-inflammatory	Protein hydrolysates, peptides, and fermented RSM	In vitro, cell models, and animal models	ACE-inhibitory peptides LY, RALP, and GHS reduce NO and pro-inflammatory cytokines in LPS-stimulated RAW264.7 cells and in the plasma of hypertensive rats 2.2. Napin-derived Thr-Leu (TL) shows anti-inflammatory and barrier-protective effects in the Caco-2/RAW264.7 coculture model.	[90,92]
Antihypertensive	ACE-inhibitory peptides and phenolic extracts	Animal models and in vitro enzyme assays	Multiple peptides show strong ACE/renin inhibition in vitro and significantly reduce systolic blood pressure in spontaneously hypertensive rats after oral intake.	[88,104,105,106,107,108,109]
Antimicrobial	Napin and cruciferin-derived peptides	In silico and in vitro	In silico hydrolysis identifies multiple candidate antimicrobial peptides from rapeseed proteins; purified napin shows clear antibacterial activity in vitro, while cruciferin does not, despite docking-based predictions. In silico proteomic profiling of RSM identifies rare predicted antibacterial peptides, highlighting the need for experimental confirmation.	[48,98]

Overall, the current evidence for the antimicrobial functionality of RSM proteins and derived peptides is still limited and should be viewed as hypothesis-generating. Napin clearly exhibits in vitro antimicrobial activity against selected bacterial and fungal targets, and rapeseed proteins are rich precursors of theoretically active AMPs, but most sequences from cruciferin, napin, and oleosin remain untested beyond bioinformatics pipelines. While predicted AMPs are generally non-toxic and many are predicted non-allergenic, their actual antimicrobial spectrum, potency, stability during processing and storage, behavior in complex food systems, and detailed mechanisms of action must be assessed systematically (Table 2).

### 4.4. Oil Binding, Nanocarriers and Delivery Matrices

Interactions between proteins and lipids are driven by hydrophobic attraction between aliphatic chains of fatty acids and the nonpolar side chains of certain amino acids. According to Zhang, et al. [110], rapeseed protein isolates obtained by alkaline extraction and acid precipitation showed substantial oil-holding capacities, reflecting the contribution of surface-exposed hydrophobic residues and residual lipid protein associations at the particle level. Protein-enriched fractions generally display higher oil absorption compared with unfractionated meal, likely due to their higher protein contents and accessible hydrophobic domains [37,58]. Conversely, ethanol pretreatment of RSM may reduce protein solubility by promoting protein-protein interactions and partial aggregation through dehydration effects, thereby modifying the oil absorption capacity of the extracted protein fractions [111]. Processing steps that induce denaturation of cruciferin and napin, such as alkaline solubilization followed by isoelectric precipitation, might increase surface hydrophobicity through protein unfolding and partial structural rearrangement, thus favoring oil binding in rapeseed protein systems [69,110]. As a result, the high oil-holding capacities of cruciferin, napin, and oleosins enable them to bind and immobilize lipids and to stabilize oil-in-water emulsions, where cruciferin forms stiff viscoelastic interfacial films, while oleosin acts as a powerful natural emulsifier with antioxidative effects, reducing coalescence and retarding lipid oxidation [51,112,113] (Figure 4).

Beyond their role in fat binding and emulsion stabilization, RSM protein fractions, particularly napin and cruciferin, are emerging as promising binding substrates for bioactive and recombinant proteins in delivery systems. Recently, a study by Arnecke, et al. [114] demonstrated that high-purity napin and cruciferin can be obtained by integrated cation and anion-exchange chromatography, yielding well-defined fractions suitable for pharmaceutical and nutraceutical applications. Their hydrophobic and charged domains support robust non-covalent interactions with various cargo molecules, including drugs, peptides, and potentially therapeutic proteins [115,116]. For example, Wang, et al. [117] engineered acylated rapeseed protein isolates into stable nanogels with a hydrophobic core and hydrophilic shell that encapsulated hydrophobic drugs such as curcumin with very high efficiency and enhanced in vitro anticancer activity, illustrating both binding capacity and protective effect of rapeseed-based carriers [117,118]. Studies on protein-based nanocarriers further show that storage globulins structurally related to cruciferin (legumin), can encapsulate and deliver therapeutic proteins and enzymes, indicating that rapeseed globulins could be adapted for similar recombinant protein adsorption strategies. Taken together, the scalable purification of napin and cruciferin demonstrated encapsulation of small-molecule drugs and broader evidence on protein-based nanoparticles for enzyme and protein delivery support the view that RSM protein fractions are versatile, sustainable platforms for binding and delivery of recombinant therapeutics, while also serving as effective oil binding and emulsion structuring ingredient (Figure 4).

## 5. Protein–Phenolic Interactions and Their Impact on Structure–Function Relationships

Building on the techno-functional properties described in Section 4, this section examines how specific molecular interactions between RSM storage proteins and endogenous phenolic compounds underpin these behaviors. In particular, complexes between cruciferin or napin and sinapic acid derivatives can alter protein solubility, surface hydrophobicity, interfacial activity, and digestibility, thereby modulating the emulsifying, foaming, and antioxidant properties observed at the macro-scale.

### 5.1. Functional Synergy Among Phenolics and Bioactive Peptides

Protein and phenolic interactions are increasingly recognized as major determinants of the structural stability, techno-functionality, and bioactivity of RSM-derived compounds. In RSM and other oilseed matrices, phenolic compounds and protein-derived peptides act jointly to shape both antioxidant and metabolic effects [22,100]. Functional interactions among RSM bioactive compounds appear to enhance both antioxidant and metabolic effects, particularly through the interplay of phenolic compounds and protein-derived peptides. A recent study revealed that phenolic-rich RSM extracts dominated by sinapic acid derivatives and flavonoids show markedly stronger α-glucosidase inhibition than the individual compounds [34], highlighting potential synergistic effects relevant to diabetes, cardiovascular disease, and neurodegeneration [38,71]. In parallel, rapeseed proteins represent a rich source of bioactive peptides, with predicted antioxidant, antihypertensive, antimicrobial, and immunomodulatory properties [42,95]. Interactions between peptides and residual phenolic compounds may further enhance cellular antioxidant and immunomodulatory responses, suggesting that peptide and phenolic complexes can shift bioactivity from chemical assays toward more biologically relevant cell-based systems [13,31]. However, many of these effects remain primarily supported by in vitro and computational studies, and confirmation through standardized in vivo and human clinical investigations is still limited.

### 5.2. Molecular Mechanism of Protein–Phenolic Binding in Rapeseed Systems

#### 5.2.1. Non-Covalent Interactions and Structural Modulation

At the molecular level, it has been shown that the storage proteins, particularly cruciferin and napin, form complexes with sinapic acid derivatives and related phenolics via reversible non-covalent and, under appropriate conditions, irreversible covalent binding [22,119,120]. At low to moderate phenolic-to-protein ratios, hydrophobic interactions, hydrogen bonding, and electrostatic attraction drive non-covalent associations, inducing moderate conformational rearrangements, altering surface hydrophobicity and thermal stability, and thereby affecting emulsification, foaming, solubility, and digestibility [11,21,121]. In rapeseed systems, phenolics such as sinapic acid and sinapine can markedly reduce solubility and impair gelation when present at high levels, whereas partial phenolic removal or controlled retention can enhance interfacial properties and in vitro digestibility [11,119,122,123]. Similar effects have been reported in other plant protein polyphenol systems, including *Pisum sativum*, *Oryza sativa*, *Cannabis sativa*, and *Fagopyrum esculentum*, where controlled non-covalent complex formation with polyphenols improves emulsifying and foaming properties and often increases in vitro digestibility and antioxidant capacity [124,125,126].

#### 5.2.2. Covalent Oxidative Interactions and Cross-Linking

**Figure 5:**
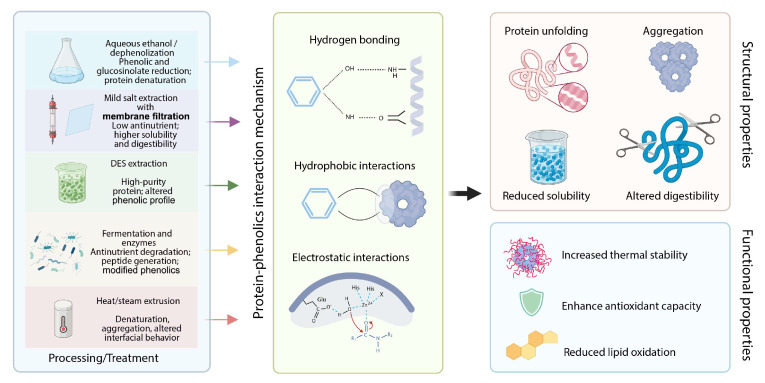
Schematic overview of protein–phenolic interactions in RSM systems and their effects on structural and functional properties. Different extraction, dephenolization, fermentation, and thermal treatments influence interactions between rapeseed proteins and phenolic compounds. Non-covalent interactions, including hydrogen bonding, hydrophobic interactions, and electrostatic attraction, alter protein structure, solubility, digestibility, and interfacial properties, while oxidative conditions may promote covalent cross-linking and aggregation. These structural changes ultimately affect the techno-functional and bioactive properties of RSM-derived ingredients, including thermal stability, antioxidant activity, and lipid oxidation behavior. This figure was created withBioRender.com (https://www.biorender.com/).

Under oxidizing or strongly alkaline conditions, phenolic moieties can be converted into reactive quinones, which readily form covalent adducts with nucleophilic amino acid side chains. This process reduces the availability of free amino and sulfhydryl groups, promoting intermolecular cross-linking, and shifts the protein secondary structure toward more β-sheet and random-coil conformations [119,120,127]. In cruciferin-rich systems, these modifications may favor aggregation, decreased solubility, and enhanced gelation and may reduce protein digestibility in some systems, although covalent complexes can also exhibit enhanced antioxidant activity and polyphenol stability. Consequently, maintaining a balance between beneficial non-covalent associations and excessive covalent cross-linking appears critical for preserving desirable functional and nutritional properties of RSM-derived ingredients (Figure 5).

### 5.3. Effects of Antinutritional Factors and Processing Strategies on Protein–Phenolic Complexes

At higher concentrations, phenolic compounds, phytic acid, glucosinolates, tannins, and other antinutritional compounds can negatively affect protein digestibility, enzyme activity, and physiological functionality [128]. These antagonistic effects have motivated the development of selective dephenolization and fractionation strategies to decouple beneficial bioactivities from undesirable antinutritional effects. For instance, aqueous ethanolic washing and ethanol pre-treatment substantially lower phenolic and glucosinolate contents, although these treatments may simultaneously induce protein denaturation and decrease solubility (Figure 5) [11,119]. In contrast, membrane-based fractionation and milder combinations, such as ethanol–ultrasound or aqueous treatments plus ultrafiltration, preserve more native proteins and some phenolics, resulting in concentrates and isolates with high solubility and unique mechanical properties (Figure 5) [129]. Partial removal of phenolics, coupled with alkaline pH-shift, can further increase solubility, surface hydrophobicity, and emulsion stability while improving in vitro digestion of canola protein emulsions [119]. Moderate alkaline extraction (around pH 9–10) generally favors the preservation of protein structure and non-covalent complexation, whereas strongly alkaline conditions (pH ≥ 12), especially with oxygen, accelerate phenolic oxidation, and covalent cross-linking [120].

Processing and fermentation further modulate protein–phenolic interactions and downstream functionality. Betchem, et al. [130] found that solid-state and enzymatic fermentations of canola press cakes with *Bacillus subtilis* and *Saccharomyces cerevisiae* can simultaneously shift phenolic profiles, reduce sinapine and related phenolics, and increase soluble protein and peptide contents, generating fractions with lower phenolic loads and improved bioavailability and sensory properties [123,131]. Moreover, modern approaches such as magnetic-field-assisted solid fermentation further improve in vitro protein digestibility and nitrogen release by promoting disulfide bond disruption [130]. Thermal and pH-shift treatments also influence interaction behavior; for instance, short high-temperature steam injection can selectively aggregate cruciferin while maintaining napin solubility, thereby modifying interfacial and foaming characteristics in phenolic-containing systems [11,53,122]. Collectively, these findings demonstrate that extraction, dephenolization, pH-treatment, extrusion, and fermentation strongly influence the formation and stability of peptide–phenolic complexes and consequently affect the solubility, interfacial properties, and nutritional and biological potential of the resulting ingredients (Figure 5).

To better characterize the synergistic and antagonistic interactions within RSM systems, advanced analytical and modelling approaches are being layered onto traditional functional assays. Multi-endpoint antioxidant and enzyme-inhibition tests, combined with isobolographic and combination-index analyses, have been proposed to quantify interaction profiles among phenolics and peptides or intact proteins [132,133,134]. In addition, high-resolution proteomics, LC-MS metabolomics, and molecular networking approaches are being applied to identify key phenolic metabolites, glucosinolates, and bioactive peptides and to track their transformations during extraction, dephenolization, extrusion, and fermentation [35,42]. Nevertheless, direct molecular-level characterization of binding interfaces between cruciferin, napin, and dominant rapeseed phenolics such as sinapic acid and sinapine remains limited, and many mechanistic models still rely on analogies to other plant protein systems.

Seed-coat pigmentation genotype is an important but underexplored determinant of protein–phenolic interactions in rapeseed. It has been shown that the yellow-seeded *B. napus* lines, which exhibit thinner seed coats and lower total phenolic content, yield protein fractions with altered phenolic loads and interaction characteristics compared with conventional black-seeded varieties [135]. Manipulating seed-coat phenolic composition through breeding therefore represents a complementary genetic strategy for generating RSM ingredients with more predictable and favorable protein–phenolic interaction profiles. At the systems level, emerging frameworks are moving beyond single compounds, toward network-based interpretations of bioactivity. Concepts from network pharmacology, microbiome, metabolome integration, and matrix-oriented bioavailability design are being applied to polyphenol–microbiota–host interactions and multi-target physiological responses [136]. These approaches may help explain why nominally similar rapeseed ingredients, differing in the protein–phenolic architecture, can elicit divergent metabolic and microbial outcomes among individuals.

Despite this progress, substantial gaps remain in RSM bioactive compounds and peptide–phenolic complexes, making this an active research area. In vivo animal work on protein–polyphenol complexes is growing, and there is evidence for improved antioxidant status, hypoglycemic effects, and microbiota modulation in non-rapeseed systems [120,127,133], but human studies on matrix-modified rapeseed fractions, peptide–phenolic complexes, and phenolic-rich concentrates remain scarce. Furthermore, multi-omics datasets and GWAS/GBS information on phenolics and antinutrients have only partially been translated into predictive design rules for functional foods [136]. Many synergistic bioactivities attributed to peptide–phenolic complexes are still demonstrated only in chemical and cell-based assays, underscoring the need for standardized in vitro digestion protocols, in vivo studies, and human interventions before robust health claims can be made for rapeseed protein–phenolic ingredients.

## 6. Potential Application of RSM Bioactive Compounds as Functional Ingredients

**Figure 6:**
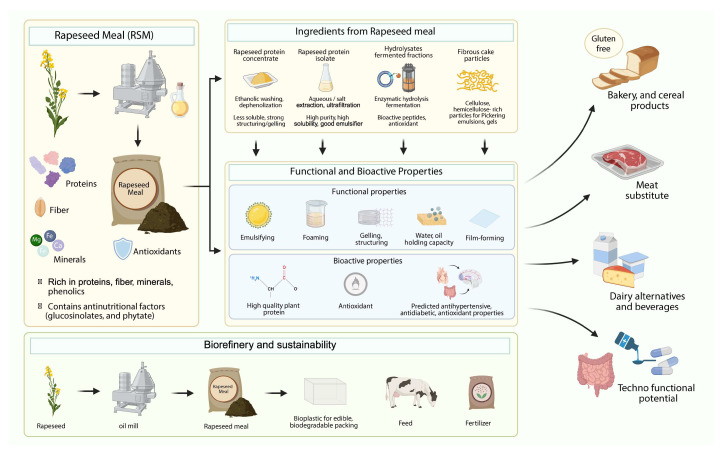
Comprehensive overview of RSM utilization as a multifunctional ingredient source with diverse food and pharmaceutical applications. This figure was created with BioRender.com (https://www.biorender.com/).

Rapeseed oil has a well-documented history of culinary use in several regions, whereas RSM has been utilized predominantly as animal feed and is recently being evaluated for human food applications [23,137]. Protein isolates derived from the RSM have been incorporated and tested in specific product categories, including wheat and gluten-free breads, plant-based beverages, yogurt-like fermented products, meat analogues, and cereal-based extrudates [21]. The following section examines emerging food-industry applications of these RSM bioactive compounds (Figure 6).

### 6.1. Potential in Cereal, High Protein Beverages, and Dair Type Products

RSM and its protein isolates have been characterized as valuable ingredients in the production of wheat and gluten-free breads, where they can enhance nutritional quality. For instance, gluten-free bread formulations supplemented with rapeseed protein isolates at low levels increase protein content and loaf volume, with a balanced amino acid profile [138]. Similarly, substituting 10% of wheat flour with RSM obtained from peeled rapeseed kernels markedly increases high quality protein, dietary fiber, and minerals, thereby improving the nutritional value and antioxidant potential [37].

Rapeseed protein-rich meal has also served as a viable component in the formulation of diverse nutritional beverages, mainly as sources of plant proteins, minerals, and phenolic antioxidant compounds [22,71]. Recently, rapeseed protein isolates obtained via mild fractionation of RSM, specifically through weakly acidic salt extraction, combined with enzymatic-assisted alkaline extraction, have been proposed for plant-based milk alternatives. These isolates offer higher solubility, lower antinutrient content, and improved digestibility, with favorable foaming and emulsifying capacities [71,139]. By employing such extraction methods followed by purification, rapeseed proteins can be formulated into beverages with acceptable sensory properties, good protein solubility, and stable emulsions [68,139]. Given their favorable amino acid profile, considerable protein content, and the presence of minerals and phenolic compounds, rapeseed protein beverages could serve as appealing options within the expanding category of plant-based milk for consumers seeking non-dairy, non-soy, cholesterol-free, lactose-free, gluten-free, nutritious, and sustainable drinks [22,68].

Furthermore, other notable food applications in which rapeseed protein and RSM-derived ingredients constitute major components include plant-based dairy products such as yogurt-like fermented products and creamy emulsions, where the foaming, gelling, and emulsifying properties of cruciferin and napin-rich fractions are utilized to enhance texture [21,22,68]. Fermented RSM and its hydrolysates, rich in phenolic compounds and short peptides, may substantially enhance the antioxidant capacity and protein digestibility, as well as generate bioactive peptides with reported anti-hypertensive and antioxidant activities in vitro and in animal models, while also modulating gut microbiota composition [42,95,129]. Nevertheless, systematic studies on their impact on probiotic viability, lactic acid bacteria growth, and microstructural stability in fermented dairy or plant-based products remain limited. In addition, the role of antinutritional factors such as glucosinolates and phytates still requires careful management through breeding, fractionation, and fermentation [21,38]. Overall, the rapeseed-based ingredients and their fermented products are attracting increasing research interest due to their promising functional properties, high nutritional value, abundant potential bioactive peptides, and role in sustainable food systems [23]. Yet, challenges remain in managing bitterness and off-flavors associated with phenolics and sulfur compounds and in fully characterizing the impacts on probiotic growth, microstructure, and long-term consumer acceptance.

### 6.2. Potential in Extrusion-Based Foods

RSM has also been shown to be an important functional ingredient in the formulation of extrusion-based food products. For instance, with the aid of optimized low- and high-moisture extraction conditions, rapeseed press cake has been successfully incorporated into protein fat extrudate systems to produce both high-moisture fibrous meat substitutes and low-moisture textured vegetable products [140,141,142]. Furthermore, when RSM and rapeseed press cake partially replace starch rich cereal fractions in mixed extrusion formulations, the crude protein content of the resulting extrudates can increase from about 8–21% to approximately 29–70% on a dry matter basis, due to the higher protein concentration of the RSM ingredients and the concomitant dilution of the low-protein starch components. These higher protein levels primarily reflect formulation-driven enrichment with protein and lipid-rich rapeseed fractions, rather than a universally higher intrinsic protein content of rapeseed compared with soy [37,38,140,141,143]. In addition, the residual oil fraction from RSM contributes extra lipids, while starch is simultaneously diluted and dietary fiber increased compared with cereal and soy-based extrudates [142,144]. Recent work on both low- and high-moisture extrusion highlights that the moisture content, barrel temperature profile, screw speed, and specific mechanical energy are critical levers controlling texturization, phase behavior, and the mechanical properties of rapeseed-containing meat analogues [145,146].

Numerous studies have examined the reduction of antinutritional factors in RSM during extrusion. Dry extrusion at about 150 °C inactivates myrosinase but only modestly reduces total glucosinolates unless combined with chemicals such as alkali plus ferrous sulphate and ammonia, which can then achieve reductions of roughly 6780% [145,147]. Under milder low-moisture conditions more typical of food and feed, extrusion generally lowers glucosinolates by only about 10–15% and has limited effect on sinapine and tannins, but can substantially reduce other antinutrients, including polyphenols, condensed tannins, saponins, and trypsin inhibitors and improve the microbiological safety and overall functionality of RSM and oilseed-based blends for meat analogues and snacks [123,145,148] (Figure 6).

According to Martin, Osen, Karbstein, and Emin [143], integration of RSM modifies the expansion, morphology, and texture of starch-based extrudates. Microscopic and X-ray microtomography analyses showed that adding 10–70 g RSM per 100 g starch yields extrudates with smaller air cells but higher cell density than starch controls, while higher RSM levels at around ≥120 °C could enhance sectional and volumetric expansion [143,149]. RSM-rich extrudates also exhibited a more uniform and finely porous cell structure while maintaining expansion and acceptable hardness, thus achieving textural properties comparable to those of commercial expanded cereal snacks [142,144]. Since texture is closely linked to internal cell size, smaller cells generally produce denser, less crispy, and harder extrudates, whereas larger cells yield lighter, puffed, and crispier products [150]. RSM-enriched extrudates have been reported to remain within the range associated with crispy cereal foams when temperature and moisture content are optimized [143,144,149]. Furthermore, rapeseed protein-enriched foods, such as high-moisture meat substitutes and cereal-based extrudates, can achieve good and acceptable sensory quality when extrusion conditions and product formulations are optimized to reduce bitterness from residual phenolic compounds and glucosinolates [23,75,140,150]. Rapeseed-fortified extrudate snacks and texture-modified foods, including softer protein-fortified matrices for older adults with chewing or swallowing difficulties and extruded cereal and meat analogue products, use RSM proteins to enhance both texture and nutrition. These products therefore combine favorable sensory attributes with a more balanced nutritional profile, particularly higher protein, mineral, and dietary fiber contents [140,141]. The subsequent section discusses the strategies that can be used to minimize the antinutritional factors from the RSM (Figure 6).

Despite the promising applications of RSM-derived proteins and phenolics in foods and bioproducts, their wider use remains constrained by antinutritional factors and by the need to control protein–phenolic interactions during processing. The following section, therefore, reviews processing strategies designed to reduce glucosinolates, phytic acid, and other antinutritional components while preserving or enhancing the functional properties highlighted above.

## 7. Processing Strategies to Balance Antinutritional Factors

As discussed in Section 5, pH, temperature, ionic strength, oxidation state, and enzymatic and fermentative treatments control whether cruciferin and napin form predominantly reversible, non-covalent complexes or irreversible covalent adducts with sinapic acid derivatives and other phenolics. The processing strategies below can therefore be viewed as practical levers to steer these interactions, and thus to tune solubility, functionality, and digestibility of RSM ingredients.

RSM, a compelling reservoir of bioactive compounds with significant functional properties, presents a promising avenue for valorization [22,141]. However, antinutritional factors, notably glucosinolates and phytic acid, limit RSM direct use by impairing flavor, digestibility, and mineral bioavailability. More recently, aqueous ethanol washing has been widely applied not only to reduce antinutritional factors but also to disrupt existing protein–phenolic complexes. It typically raises the protein content from 37 to 42%, lowers lipids, and reduces phenolics and glucosinolates [47,70]. At the same time, ethanol and heat promote protein denaturation and aggregation, decreasing cruciferin and napin solubility [151], lowering lysine and shifting the extractable profile towards more insoluble fractions, which reshapes functional properties [11]. Recently, a study by Đermanović, Marić, Sakač, Tomić, Dragojlović, Popović, Šarić, and Jovanov [47] has optimized dephenolization via ethanol coupled with artificial neural networks, to achieve 90% protein purity with improved solubility, emulsification, and digestibility at industrially relevant yields; however, the robustness of this approach across feedstocks remains to be demonstrated.

To balance yield, antinutritional factors removal and functionality, mild extraction strategies that preserve native protein structure and a controlled level of non-covalent protein–phenolic complexes are gaining attention. Weakly acidic salt extraction of MgCl_2_ at pH 6.5, followed by ultrafiltration, can deliver edible rapeseed protein with 40% extraction yield, low glucosinolates, phytic acid, and sinapine, favorable amino acid patterns, and better in vitro gastroduodenal digestibility, color, odor, solubility, and emulsifying or foaming properties compared with conventional alkaline isolates [139,141]. These conditions limit phenolic oxidation and extensive covalent crosslinking, thereby maintaining cruciferin and napin in more native, soluble forms, where reversible peptide–phenolic complexes can still contribute positively to interfacial properties. In addition, membrane filtration further enriches protein from 45 to 63% while removing phenols and low molecular weight solutes [16,137], offering a way to decouple strongly bound or low-molecular-weight phenolics from larger protein–phenolic assemblies while largely preserving tertiary and quaternary structure. Furthermore, short high-temperature treatment by direct steam injection selectively aggregates cruciferin while keeping napin largely soluble, leading napin to dominate interfacial and foaming behavior, illustrating how targeted processing can fine-tune the relative roles of both storage proteins in air water interfaces [121].

Emerging deep eutectic solvent (DES) systems are also being investigated. Under optimized conditions, DES-based extractions have been reported to yield protein purities up to 96% and a balanced amino acid profile [16,17]. Compared with harsh alkaline extraction, DES systems can provide high purities with less protein damage, suggesting a more controlled modulation of protein–phenolic interactions; however, their precise impact on non-covalent vs. covalent complexes, and on the retention or removal of sinapic acid derivatives, remains to be fully mapped. DES-extracted isolates often show higher Sulfur amino acid content and better solubility than strongly alkaline isolates, although optimization for scale-up, solvent recovery, and tighter control of glucosinolates and phytic acid remains to be established [16,17,47].

Furthermore, complementary biological detoxification strategies can substantially improve the functionality of RSM bioactive compounds by combining targeted enzymatic treatments with solid-state fermentation. It has been reported that the combined phytase treatment and ethanol detoxification of rapeseed protein isolates yield products with 88 to 90% protein purity, and markedly lower glucosinolates and phytic acid levels. Additionally, short-term rodent feeding studies with these protein isolates showed no adverse or toxic effects [114,152]. Nevertheless, comprehensive safety assessments, including multi-generational studies, are still needed. Additionally, solid-state fermentation with *Aspergillus niger*, or *Bacillus consortia* can decrease glucosinolates and phytic acid by 40 to 90% and increase trichloroacetic acid-soluble protein, small peptides, and total phenolics and flavonoids [18,114,121,153,154,155,156,157].

Together, enzymatic detoxification and microbial fermentation act synergistically to open cell wall structures and hydrolyze phytic acid, while fermentative microbes secrete proteases, carbohydrates, and phytases that further degrade antinutritional factors and storage proteins.These treatments, therefore, simultaneously reduce harmful protein–antinutrient complexes and create new peptide–phenolic assemblies with potentially improved bioactivity. This results in protein-rich products with improved techno-functional performance suitable for feed and potential food applications [21,158]. Overall, the integration of mild physical and chemical extraction methods with targeted biotechnology and fermentation offers a coherent processing toolbox for deliberately modulating cruciferin, napin, and phenolic complexes. This approach aligns antinutrient management with the structure–function relationships discussed in Section 5 and supports biorefinery strategies aimed at stabilizing RSM proteins and co-fractions while mitigating antinutritional constraints.

## 8. Limitations and Future Prospects

RSM is rich in bioactive substances, notably high-quality proteins, peptides, and phenolics, which show promising potential for supporting human health. In addition, these bioactive compounds are naturally concentrated in vesicle-like nanoparticles, making them an ideal vehicle for delivering health-promoting effects. For instance, buckwheat bioactive fractions display protective effects on liver function, while bioactive compounds from kidney beans stimulate short-chain fatty acid production and enhance gut microbiota [159]. However, the functional roles of RSM bioactive compounds remain underexplored. Importantly, the most reported antioxidant, antihypertensive, antimicrobial, and anti-inflammatory effects of RSM bioactive compounds are based on in silico, in vitro, or animal studies, with virtually no human clinical evidence available at present. Consequently, further studies are required to support the techno-functional and health-promoting properties of the RSM bioactive compounds.

Despite the high protein content of RSM, its balanced amino acid profile, and high levels of phenolic antioxidants and other bioactive compounds, large-scale development and production of RSM ingredients still face substantial limitations. These include variations in the composition driven by cultivar, agronomic conditions, and extraction technique. In particular, the presence of antinutritional factors, which limit flavor, protein digestibility, and nutritional bioavailability, restricts entry into high-value food and pharmaceutical industries despite higher crude protein levels and a favorable essential amino acid profile. Although conventional and molecular breeding, and genome editing strategies targeting antinutritional factors offer promising routes to develop low antinutrient genotypes while maintaining protein yield and targeted phytochemicals, future research should focus on integrating these genetic improvements with downstream processing performance and functional food applications specific to rapeseed proteins.

On the processing side, controlling protein–phenolic interaction remains a major technological challenge. The formation of protein–phenolic complexes is particularly influenced by pH shifts, temperature, ionic strength, oxidation state, and enzyme-induced structural alterations. These factors can promote excessive complexation, precipitation, or reduced digestibility over time. Advanced modern analytical techniques, such as HPLC-MS/MS and molecular simulation, should be employed to elucidate binding mechanisms in depth, conformational changes, and aggregation behavior of cruciferin and napin-rich rapeseed protein matrices interacting with sinapine, sinapic acid, and related phenolic acids. Particular attention should be directed toward identifying the specific binding sites and interaction forces between cruciferin, napin, and sinapic acid derivatives, as these mechanisms are currently poorly understood but critically influence protein solubility, emulsifying properties, digestibility, and antioxidant activity in food systems. This will provide a solid theoretical foundation for optimizing extraction, dephenolization, and fractionation conditions that balance protein functionality with phenolic recovery.

Product diversification possesses substantial potential to increase value and broaden the utilization of RSM bioactive compounds within food, nutritional, cosmetic, and pharmaceutical sectors. Rapeseed protein concentrates and isolates, with favorable emulsifying, foaming, and gelling properties, serve as promising bases for plant-based formulations that partially or fully replace animal proteins in bakery, meat substitutes, beverages, emulsified sauces, and dairy products. Functional foods enriched with standardized phenolic extracts, sinapic-acid-rich fractions, and bioactive peptide hydrolysates can be formulated to target antioxidant, anti-inflammatory, hypolipidemic, antihypertensive, and antidiabetic processes, as demonstrated mainly by in vitro and preclinical studies [38,68,160]. High-moisture extrusion and the use of fermented protein substrates are increasingly used to produce protein-fortified texture-modified foods for older adults. These processing and fermentation strategies can enhance protein solubility, improve texture and sensory quality, and contribute to longer shelf life [22,23]. Among these applications, plant-based beverages, emulsified foods, and high-moisture meat analogs currently represent the major areas requiring standardization for RSM ingredients, particularly regarding protein stability, flavor consistency, and sensory performance during storage and processing.

Beyond food, the relatively high content of sinapic acid derivatives and other phenolics in RSM, reported radical-scavenging, antimicrobial, and photoprotective activities, makes it a promising source of natural compounds for cosmetic and skincare formulations. Such extracts could be integrated into antioxidant and anti-aging skincare products that support sustainable, plant-based “green” beauty concepts. To enable standardized use of RSM-derived ingredients in high-value food and cosmetic applications, scalable and economically feasible processes for producing protein concentrates, isolates, hydrolysates, and spray-dried or freeze-dried phenolic extracts with well-defined composition and functional properties will be essential. Future studies should also prioritize the development of reproducible, food-grade fractionation pipelines for napin-rich proteins, bioactive peptide hydrolysates, and phenolic-rich extracts to improve batch-to-batch consistency and industrial applicability.

Rising global demand for sustainable alternative proteins further increases the need for positioning RSM as a versatile, high-value ingredient for food and pharmaceutical applications. Increasing consumer and industry awareness of the nutritional quality, functional properties, and antioxidant potential of RSM proteins, peptides, and phenolic fractions through scientific communications and marketing can help shift RSM beyond its current low-value roles. The future of the RSM sector depends on a multifaceted strategy that combines breeding and agronomic improvement, product advancement, and integrated biorefinery-based fractionation of proteins and phenolic compounds. Nonetheless, additional work is needed to fully optimize antinutrient management and ensure large-scale, reproducible standardization.

In conclusion, RSM is a rich source of bioactive proteins, peptides, and phenolics antioxidant that can be selectively concentrated and tailored by advanced extraction, fermentation, and fractionation technologies. As a result, RSM derived bioactive fractions show promise for improving oxidative stability, while also serving as sustainable inputs for agriculture and biotechnology. Moving forward, future research is expected to integrate plant breeding, process engineering, fermentation biotechnology, and AI-driven optimization to overcome current technical limitations and provide innovative RSM-based solutions for sustainable protein supply for human consumption.

## Data Availability

No new data were created or analyzed in this study. Data sharing is not applicable to this article.

## Abbreviations

The following abbreviations are used in this manuscript:

RSM	Rapeseed meal
BCAAs	Branched-chain amino acids
DPPH	1,1-Diphenyl-2-picrylhydrazyl
MAPK	Mitogen-activated protein kinase
JNK	N-terminal kinase
AP-1	Activator protein-1
NF-κB	Nuclear factor kappa-light-chain-enhancer of activated B cells
KEAP1	Kelch-like ECH-associated protein 1
NRF2 (Nrf2)	Nuclear factor erythroid 2-related factor 2
ARE	Antioxidant response element
EDTA	Ethylenediaminetetraacetic acid
DES	Deep eutectic solvent
SCFA	Short-chain fatty acids
HPLC-MS/MS	High-performance liquid chromatography tandem mass spectrometry
UPLC	Ultra-performance liquid chromatography
Caco-2	Human intestinal epithelial cell line
HT29	Human colon adenocarcinoma cell line
RAW264.7	Murine macrophage cell line RAW 264.7
LPS	Lipopolysaccharides
kDa	Kilodalton
GWAS	Genome-wide association studies
GBS	Genotyping-by-sequencing
